# Inception of a pediatric cancer caregiver support group guided by parental needs

**DOI:** 10.1002/cnr2.1469

**Published:** 2021-06-06

**Authors:** Vasudha N. Rao, Rajeshwari Anantharaman Rajeshwari, Revathi Rajagopal, Michelle Normen

**Affiliations:** ^1^ Department of Pediatric Oncology Cytecare Cancer Hospitals Bangalore India; ^2^ Department of Psycho‐oncology Services Cytecare Cancer Hospitals Bangalore India; ^3^ Department of Psycho‐oncology HCG Cancer Hospitals Bangalore India

**Keywords:** Emotional Needs, Empowering Caregivers, Holistic Care, Informational Support, Pediatric‐Oncology Caregiver Support, Psycho‐Oncology in Childhood Cancer

## Abstract

**Background:**

Survivorship in childhood cancers has steadily improved and increased the need for caregivers to provide a longer duration of care both in the hospital and at home. Involving parents and caregivers to voice their unmet needs could significantly impact and direct the institution of support groups.

**Aims:**

To ascertain the need for a pediatric caregiver support group based on a survey that explored the unmet needs of caregivers of children with cancer.

**Methods:**

Caregivers of pediatric patients (*n* = 17) undergoing treatment at the hospital were requested to complete our Pediatric Caregiver Psycho‐social Needs Survey. The survey encompassed questions on different aspects of caregiving and the caveats felt by the caregivers. The needs were categorized into seven main domains (physical, emotional, family‐related issues, spiritual, social, logistics, and information) that focused on understanding the importance and the perceived level of professional support that was expected. The data was analyzed using SPSS.

**Results:**

The most often reported needs were (i) emotional concerns with the majority reporting fear (58.8%), (ii) logistics‐related needs for play/art‐based activities (58.8%), (iii) informational needs focusing on understanding diagnosis/prognosis (47.1%), side‐effects of treatment and physical changes (41.2%). Family‐related needs escalated when caregivers (23.5%) looked after other ill family members at home. Caregivers (23.5%) also reported Spiritual concerns suggesting the need for religious/spiritual support in the hospital. Majority of caregivers (82.4%) expressed interest to be part of a pediatric caregiver support group. However, professional support sought for was much lesser compared to the percentage of needs/concerns expressed.

**Conclusion:**

Our study highlighted the unmet needs of caregivers which included emotional, logistics‐related needs, and concerns about information. Hence, the goal is to provide a unified platform through a support group that holistically can address needs and empower caregivers.

## 1 INTRODUCTION

The diagnosis of pediatric cancer has a multifold psychosocial effect not just on the child in question but the entire family. In particular, challenges faced by the caregivers encompass financial, social, and emotional domains and persist even after the completion of treatment. The experience of caregiving for patients with cancer has been described as “intense, episodic, and challenging.”.^1^

In the pediatric oncology setting, demands of caregiving are protean and ranges from medical care (management of symptoms, timely administration of medication, care of central venous access) to balancing the logistics of transport, accommodation and battling the cultural stigma of a cancer diagnosis. The impact of psychosocial aspects on caregivers most often the parents cannot be stressed enough. Many caregivers experience to name a few, a multitude of complex emotions, have to assimilate information about the illness, be providers to their healthy children and provide round the clock care to the patient.^2^, ^3^, ^4^ A study by Kearney et al. on Standard of Psychosocial care for parents of children with cancer high‐lighted multiple studies which reported that both parents were likely to exhibit significant distress which sometimes lasted up to 5 years post‐diagnosis. The negative impact of parental distress resulted in poorer quality of life (QOL), emotional, and physical health factors, dysfunctional family functioning and greater marital distress. Numerous factors have also emerged as indicators of risk for parental maladjustment resulting in increased caregiver burden. They can be classified into socioeconomic factors like lower household income, lower level of education, lack of employment; pediatric disease factors such as relapse, treatment severity/risk or poorer child's functional impairment or physical symptoms; prior traumatic life events, and prior parent psychiatric treatment.^5^

An extensive literature base supports that caregiver interventions have the potential to improve caregiver QOL and emotional well‐being^6^, ^7^ for adult patients with cancer and alleviate distress in caregivers of pediatric patients with cancer.^8^ In India to be specific often owing to the overwhelming burden of clinical commitments, clinicians may not always be able to devote time to address the psychosocial concerns of caregivers.

Thus, support groups could become a platform to address common concerns, develop skills for caring, identifying red flags, promoting self‐care, raising awareness and foster a sense of togetherness among affected families. In a country like India where the financial burden of treatment often takes over the other problems, due importance, needs to be given to the psychosocial needs of the caregiver. However, the assumption of parental needs by the clinical team can sometimes be a case of “smoke and mirrors”. To obviate this bias, we attempted to channelize the needs of parents through a formal survey and direct the results towards the creation of a caregiver support group.

## 2 METHODS

This was a cross‐sectional study that used a convenience sample. We included only primary caregivers of our pediatric cancer patients aged 3–15 years who were on follow‐up with the pediatric oncologist at our center between July and August 2019. Information on demographics (age, gender, occupation, relationship with patient) were also collected along with a question eliciting their willingness to attend a pediatric caregiver support group.

### 2.1 Pediatric caregiver psychosocial needs survey

**FIGURE 1:**
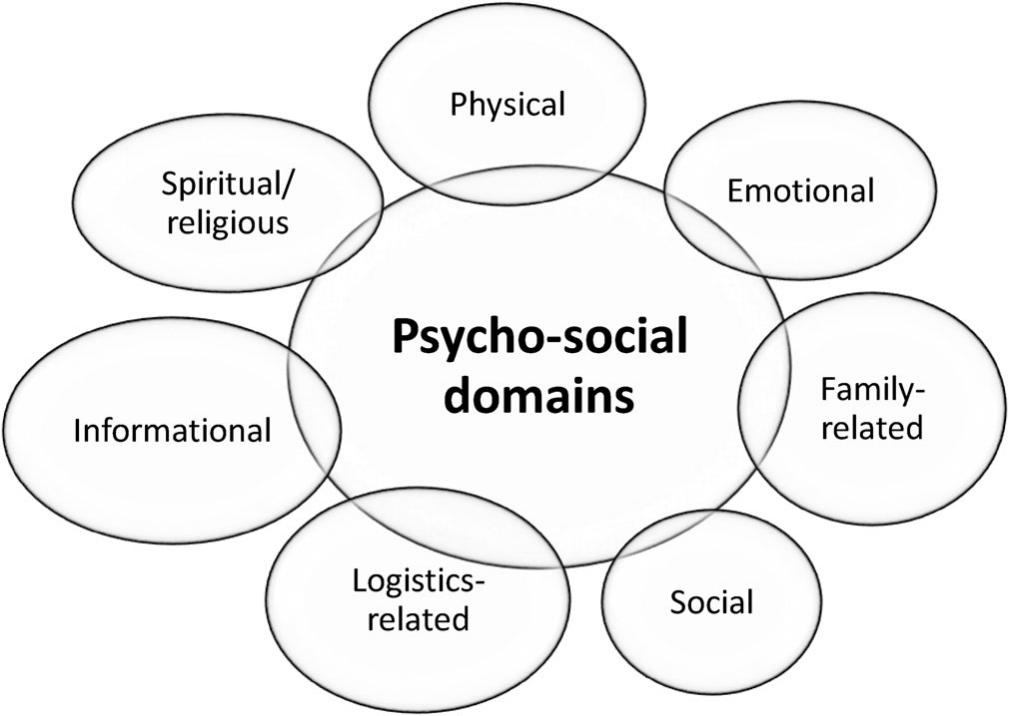
Domains assessed on the pediatric caregiver psycho‐social needs survey

The survey was developed based on a review of literature^9^, ^10^, ^11^ and domains of the NCCN (National Comprehensive Cancer Network) Distress Thermometer. Once the survey questionnaire was formulated it was given for face validity to a few experts (medical oncologist, pain and palliative care physicians, psycho‐oncologists) in the hospital. The necessary changes were made with regard to number of items and wording before it was finally given to the caregivers. The preliminary survey contained a domain on financial concerns, but after a repeat discussion we felt financial concerns by default would be a concern and require professional assistance and hence omitted the domain. The final survey contained 33 items which had seven domains and were to be answered on a 5‐point Likert scale to understand (a) level of importance (as per the caregiver) and (b) need for professional assistance. The seven domains included physical concerns, emotional concerns, family‐related concerns, social concerns, logistics‐related concerns, informational concerns and spiritual/religious concerns **(**Figure 1 **)**.

### 2.2 Study procedure

The 33‐item survey was distributed to caregivers in the pediatric out‐patient (OPD) clinic of the hospital after obtaining verbal consent. Most of the caregivers were well‐versed in English and completed the survey and the demographic detail sheet on their own. The remaining were helped by the psychologist/translator as they were more comfortable in the local vernacular (Kannada, Hindi, and Tamil) and one of international nationality (Arabic). The survey took about 10 min to complete and were returned to either the oncologist or psychologist upon completion.

### 2.3 Data analysis

The data were analyzed using SPSS v 20.0. We grouped the demographics (caregiver age, gender, occupation, and if they were the primary caregivers) and domains using descriptive statistics as frequencies and proportions.

## 3 RESULTS

We approached a total of 20 caregivers attending the pediatric oncology OPD, of which 17 consented to complete the survey. The remaining declined due to other planned engagements. Based on those included we found that almost all participants were in the age group of 24–47 years and were parents (94.1%) of the patient. More than half of our caregivers were female and the mothers (52.9%) of the patients and majority of them were homemakers (29.4%) **(**Table 1 **)**. Majority of our participants (82.4%) also reported interest to be part of the support group.

**TABLE 1:** Sociodemographic characteristics of caregivers of pediatric patients with cancer visiting the out‐patient department of a private tertiary cancer hospital (July–August, 2019)

Patient characteristics	*N*	(%)
Total	17	(100.0)
Sociodemographic details		
Age (in years)		
18–40	10	(58.8)
41–60	7	(41.1)
Gender		
Male	8	(37.9)
Female	9	(62.1)
Relationship with patient		
Mother	9	(52.9)
Father	7	(41.2)
Uncle	1	(5.9)
Primary caregiver		
Yes	16	(94.1)
No	1	(5.9)
Occupation		
Homemaker	5	(29.4)
Farmer	3	(17.6)
Businessman	3	(17.6)
Driver	1	(5.9)
Manager	2	(11.8)
IT professional	2	(11.8)
Private employee	1	(5.9)

Abbreviation: IT, Information‐technology.

Of the seven domains, the most frequently reported concerns were pertaining to emotional concerns. In this domain the most commonly reported feelings were fear (58.8%), worry (52.9%), sadness and uncertainty (47.1%) and difficulty accepting child's illness (41.2%). However, less than half of the caregivers who reported distress sought professional assistance in each of these domains; fear (17.6%) worry (17.6%), sadness (23.5%), and uncertainty (11.8%) and difficulty accepting child's illness (17.6%). The other concerns in this domain that were reported were helplessness (23.5%) and difficulty in adjusting to the new routine (17.6%). Although reported by a minority, all of them felt the necessity for professional assistance. With regard to issues pertaining to puberty and adolescence, only a minority (11.8%) reported it as concern, however the need for professional assistance (17.6%) was higher.

The next most reported domain was that of logistic concerns. Majority of caregivers reported the need for the hospital to have art based/play activities for the children (58.8%) while only (5.9%) reported requiring professional assistance on the same. Another major concern reported was on Academics/Education of the child (41.2%) although only (5.9%) required assistance. Few parents also reported the need for additional opportunities to discuss with the doctor/treating team (17.6%), but the need for professional assistance was lesser (11.8%). Logistic concerns reported on accommodation (11.8%), transportation (17.6%), need for a translator (11.8%) and issues with child care (23.5%), although were reported by a minority were all found requiring equal need for professional assistance.

In the domain of informational needs, we found that majority caregivers reported concerns with accessing information about the illness (47.1%) and more than half (29.4%) expressed need for professional assistance. The next most reported concern was regarding the physical changes endured by the child during treatment (41.2%) for which the need for professional assistance was intently expressed (41.2%). Caregivers also reported requiring information on benefits and side‐effects of treatment (41.2%) and nutrition (41.2%) although only (23.5%) and (35.3%), respectively, reported needing professional assistance.

In the spiritual domain the concerns comprised the need for a prayer room (17.6%) and need for the availability of a spiritual/religious person in the hospital (23.5%). However, the expressed need for professional assistance was higher 23.5% and 29.4%, respectively.

Among the social and family domains, caregivers reported issues pertaining to talking about cancer in social situations (35.3%), experiencing a lack of social support (29.4%) and encountering difficulties handling health‐related issues among other family members (23.5%). We found that the need for professional assistance sought was lower in these domains. On the domain on physical concerns, the need for rest and sleep were reported (23.5%) although the professional assistance needed was lower.

## 4 DISCUSSION

Support groups have been defined as “groups of people with common experiences and concerns who provide emotional and moral support for one another.” In the context of pediatric cancers and other childhood chronic diseases, support groups also combine comprehensive education in order to support the caregiver's informational needs.^12^ For parents of children with special needs, research has shown that common themes or experiences are feelings of intense anxiety, depression, loneliness, and hopelessness.^13^ In addition to the responsibility of parenting, families and parents often find themselves inadequately skilled to handle a complex world of medications, symptoms, procedures, and investigations. In parallel with the cure rates in pediatric cancers, there is an increasing need to address the issues of growing number of families as well. More often than not, the entire focus of care and support is on the physical concerns of the child, aiming at cure. A study conducted by Schmid et al. reported experiences of a support group that was initiated by parental unrest in the pediatric bone marrow transplant unit which however, did not operate with a prefunctional needs survey. The study showed that understanding of the main, or previously unrecognized, family issues through the support group resulted in a better outcome for the patients and caregivers.^14^

In our study the most frequently reported concerns were pertaining to emotional issues, concerns with logistics and informational support. The emotional concerns that were recounted most commonly in our cohort were fear, anxiety and uncertainty which are similar in theme to other studies. An explorative study done by Carlsson et al. which assessed 15 parents for psychological distress reported “An unfamiliar and frightening situation during treatment and adjusting to the new situation” echoing the feeling of uncertainty and acceptance. Another important theme that emerged in their study was “emotional struggles after end of curative treatment and transitioning back to life as it was before the diagnosis”.^15^ In another survey‐based study done by Rajajee et al. depression was reported by 100% of caregivers who were the mothers of the children.^16^

Although a majority of caregivers reported concerns in the emotional domain, only a small percentage asked for professional assistance. This pattern was observed in almost all the other domains of the survey. We hypothesize that the reason for this discrepancy is the stigma associated with seeking psychological support and this could be as a result of public stigma and self‐stigma.^17^

The second most frequently reported domain of concern in our cohort was that of logistical‐related support, in particular play/activities for the children and need for academic support. However again only a minority requested professional assistance. Chari et al. reported that the benefits of play therapy lie in better illness adjustment and general mental well‐being, enhanced coping, and normalization.^18^ When encountered with a life‐threatening illness like cancer, caregivers may not expect activity‐/play‐based therapy to be provided by the hospital and instead focus entirely on the clinical care. This may explain the discrepancy between the concern expressed and the need for help observed in this domain.^19^ Similarly, school and academics take a back seat during treatment for various reasons including fear of infection, sociocultural stigma, and absenteeism due to side‐effects. Creating awareness and building on improving the liaison between hospital, home and school may result in long‐term benefits to the child with cancer.^20^, ^21^ A study by Tsimicalis et al. reported that school absenteeism was the most commonly reported productivity loss in children with cancer.^22^

The third significant concern voiced in our caregiver cohort was that of informational needs. Studies on difficulties in establishing effective communication with health care staff and parents with unmet informational needs have been documented for families of children with cancer in South Africa, Kenya, Turkey, and Indonesia.^23^, ^24^, ^25^ Levine et al. conducted a study to elicit pediatric oncology patient and parent perceptions of early cancer communication to establish whether informational needs were met. Contrary to our survey their study showed that a greater percentage of participants reported “a lot” of discussion about the physical impact of cancer compared with impact on QOL or emotional impact. Further, they also identified that patients and parents perceive less frequent communication regarding the emotional impact of cancer therapy compared with physical impact or the impact on QOL.^26^ Notably in a landmark study done by Jatia et al. showed that provision of a holistic support group (encompassing, financial, psychosocial, emotional, informational support) reduced the annual rate of abandonment from 20% in 2009 (basic support) to 10.4% and 5.2% in 2010 and 2011 (limited support) and has been consistently between 3% and 6% from 2012 to 2016 (enhanced support).^27^

In our cohort, nearly a third of the caregivers expressed the need for availability of spiritual help and an equal number also expressed interest in professional assistance for the same. A meta‐analysis done by Gonçalves et al. showed that religious and spiritual interventions play a significant role in reduction of stress and depression.^28^ What is noteworthy is that, families that had spiritual support were stronger and displaced resilience.^16^

### 4.1 Implications of the study

Our study has direct implications on practice. First, to the best of our knowledge this is a first of a kind study conducted in India that used the inputs of caregivers to constitute a support group. The domains that were highlighted in the study suggested for the inclusion of multi‐disciplinary teams to aid in providing holistic psychosocial support. The pediatric caregiver support group (titled *“Disha”*‐meaning direction [in Hindi]) was initiated as a result of this study. Since then, we have had eight group meetings which have addressed caregiver concerns on emotional issues, information relating to childhood cancer and treatment, nutrition, supportive care needs, etc. The actual benefits of the support group as perceived by the caregivers are a focus of interest and would be a follow‐up to this study. Second, the reporting of emotional concerns as a major concern has made us sensitive to discuss and deliberate over psychological issues faced by caregivers and address the same in the support group meetings. This has also facilitated in disseminating evidence‐based information and encourage families to seek professional psychosocial support when required and thereby help reduce stigma. Third, on a larger platform the support group could facilitate awareness building in schools and for teachers with regard to managing childhood cancers in order to warrant successful re‐entry of children post completion of treatment.

This study is also not without limitations with regard to the sample, as the primary focus was to ascertain psycho‐social needs of caregivers and their interest to join a support group. Since, the intent was to start *Disha*‐Pediatric Caregiver Support Group as part of Childhood Cancer awareness month, the time frame for data collection was short.

In conclusion, we understand the there is a need for taking into consideration the concerns of caregivers to tailor make a support group. This study highlighted the needs of caregivers which were emotional, logistics related and concerns pertaining to informational needs. Hence, the real challenge is on how we can channelize support and empower caregivers using evidence‐based information to increase awareness and thereby provide adequate psychosocial support.

## CONFLICT OF INTEREST

The authors have no conflict of interest to declare.

## AUTHOR CONTRIBUTIONS

All authors had full access to the data in the study and take responsibility for the integrity of the data and the accuracy of the data analysis. *Conceptualization*, R.A., R.R., M.N.; *data curation*, R.A., R.R., M.N.; *formal analysis*, R.A., R.R., M.N.; *funding acquisition*, R.A., R.R., M.N.; *investigation*, R.A., R.R., M.N.; *methodology*, R.A., R.R., M.N.; *project administration*, R.A., R.R., M.N.; *resources*, R.A., R.R., M.N.; *software*, R.A., R.R., M.N.; *supervision*, R.A., R.R., M.N.; *validation*, R.A., R.R., M.N.; *visualization*, R.A., R.R., M.N.; *writing‐original draft*, R.A., R.R., M.N.; *writing‐review & editing*, R.A., R.R., M.N.

## ETHICAL STATEMENT

An approval was obtained from the Head of Medical Services of our hospital prior to conducting this study. Completion of the survey was considered as consent to participate in the study.

## Data Availability

The datasets generated during this study are not publicly available to protect patient confidentiality but are available from the corresponding author on reasonable request.
